# Nomogram to identify severe coronavirus disease 2019 (COVID-19) based on initial clinical and CT characteristics: a multi-center study

**DOI:** 10.1186/s12880-020-00513-z

**Published:** 2020-10-02

**Authors:** Yixing Yu, Ximing Wang, Min Li, Lan Gu, Zongyu Xie, Wenhao Gu, Feng Xu, Yaxing Bao, Rongrong Liu, Su Hu, Mengjie Hu, Chunhong Hu

**Affiliations:** 1 Department of Radiology, The First Affiliated Hospital of Soochow University, No.188, Shi Zi Street, Suzhou, 215006 Jiangsu China; 2 Department of Radiology, The Affiliated Infectious Diseases Hospital of Soochow University, Suzhou, 215000 China; 3 Department of Radiology, The Fifth People’s Hospital of Wuxi, Wuxi, 100191 China; 4 Department of Radiology, The First Affiliated Hospital of Bengbu Medical College, Bengbu, 233004 China; 5 Department of Radiology, The First People’s Hospital of Taicang, Suzhou, 215400 China; 6 Department of Radiology, The First People’s Hospital of Suqian, Suqian, 223800 China

**Keywords:** COVID-19, Pneumonia, Tomography, X-ray computed, Nomogram

## Abstract

**Background:**

To develop and validate a nomogram for early identification of severe coronavirus disease 2019 (COVID-19) based on initial clinical and CT characteristics.

**Methods:**

The initial clinical and CT imaging data of 217 patients with COVID-19 were analyzed retrospectively from January to March 2020. Two hundred seventeen patients with 146 mild cases and 71 severe cases were randomly divided into training and validation cohorts. Independent risk factors were selected to construct the nomogram for predicting severe COVID-19. Nomogram performance in terms of discrimination and calibration ability was evaluated using the area under the curve (AUC), calibration curve, decision curve, clinical impact curve and risk chart.

**Results:**

In the training cohort, the severity score of lung in the severe group (7, interquartile range [IQR]:5–9) was significantly higher than that of the mild group (4, IQR,2–5) (*P* < 0.001). Age, density, mosaic perfusion sign and severity score of lung were independent risk factors for severe COVID-19. The nomogram had a AUC of 0.929 (95% CI, 0.889–0.969), sensitivity of 84.0% and specificity of 86.3%, in the training cohort, and a AUC of 0.936 (95% CI, 0.867–1.000), sensitivity of 90.5% and specificity of 88.6% in the validation cohort. The calibration curve, decision curve, clinical impact curve and risk chart showed that nomogram had high accuracy and superior net benefit in predicting severe COVID-19.

**Conclusion:**

The nomogram incorporating initial clinical and CT characteristics may help to identify the severe patients with COVID-19 in the early stage.

## Background

In December 2019, coronavirus disease 2019 (COVID-19) broke out in Wuhan City, Hubei Province of China [1]. Since then, the number of confirmed COVID-19 cases has increased rapidly. As of May 12, 2020, China has reported 84,458 confirmed cases and 4644 deaths. Globally, as of 3:12 pm CEST, 13 July 2020, there have been 12,768,307 confirmed cases of COVID-19, including 566,654 deaths, reported to WHO [2]. At present, COVID-19 is an emerging, rapidly evolving situation. COVID-19 has become a pandemic in the world and posed a great threat to global health [2].

The most common clinical symptoms are fever or cough in addition to other non-specific symptomatology including headache, sore throat or fatigue [1, 3]. A small number of patients may have diarrhea or dyspnea and even relatively asymptomatic [4]. Chest CT plays a vital role in the early detection and disease evaluation of COVID-19 [1, 5]. Typical CT imaging features of COVID-19 include bilateral, multifocal and peripheral ground-glass opacities (GGOs), with or without local consolidations [6, 7]. Most of patients showed multilobar involvement and pneumonia was more frequent in the lower lobes or posterior part of the lung [1].

According to Wu et al. [8] study, the majority of patients with mild and moderate diseases had a good prognosis, but the mortality rate of critical patients was high. Wu et al. [8] reported that the case-fatality rate was 49.0% among the critical COVID-19 cases in China. At present, the key of treatment is to prevent mild and moderate disease from progressing to severe or critical disease. Therefore, it is of great significance to identify the severe or critical patients and take active intervention measures in the early stage. Early identification of severe or critical patients facilitated appropriate supportive care and promptly access to the intensive care unit (ICU) if necessary [9].

In this study, we analyzed the clinical and CT imaging characteristics of 217 initially admitted patients infected with COVID-19. Independent risk factors associated with severe or critical COVID-19 were identified. A nomogram was developed and validated to predict the severe COVID-19 in the early stage of disease course.

## Methods

### Patients

**Fig. 1:**
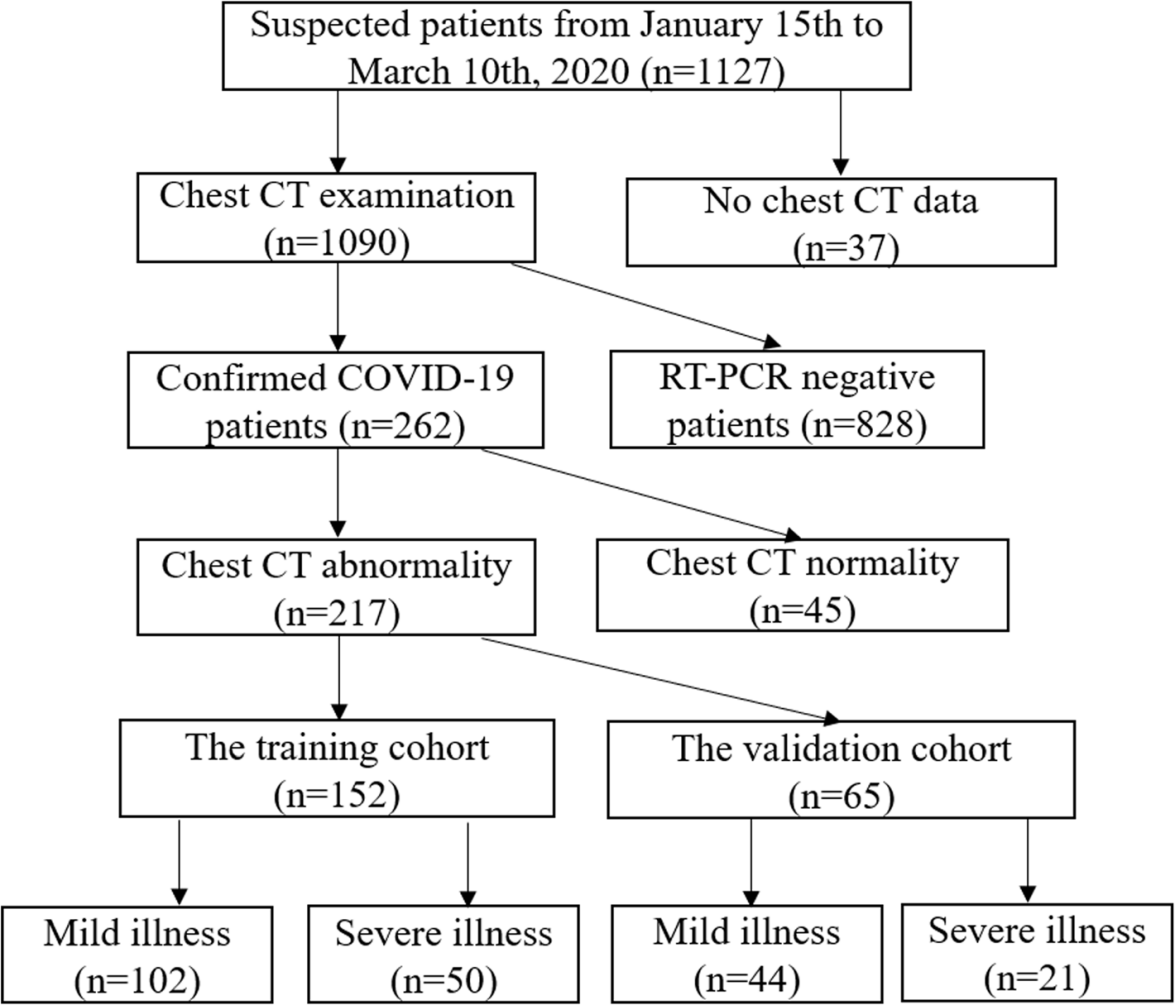
Flowchart of the study population. RT-PCR: real-time reverse transcriptase polymerase chain reaction. COVID-19: coronavirus disease 2019

Ethical approvals by our institutional review boards were obtained for this retrospective study, and the need to obtain informed consent was waived. One thousand one hundred twenty-seven suspected patients were consecutively enrolled from January 15th to March 10th, 2020 in several hospitals in Jiangsu and Anhui provinces of China. Two hundred sixty-two patients were hospitalized and had confirmed COVID-19 via laboratory testing with real-time reverse transcriptase polymerase chain reaction (RT-PCR) of respiratory secretions. A total of 217 COVID-19 patients (127 males and 90 females, mean age 46 years, age range 6–86 years) with chest CT abnormality were included in our study (Fig. 1). According to the guidelines for the diagnosis and treatment of COVID-19 (trial version 7) developed by the National Health Committee of the People’s Republic of China [10], confirmed patients are divided into mild, common, severe and critical types (Supplementary material). According to the clinical severity, the patients are divided into mild illness group (mild and common types) and severe illness group (severe and critical types) during the follow-up (Supplementary material). Of the 217 patients with COVID-19, 146 cases were in the mild group and 71 cases were in the severe group. According to a ratio of 7:3, patients were randomly assigned to the training cohort (102 mild cases and 50 severe cases) and validation cohort (44 mild cases and 21 severe cases).

### Follow-up

All patients were followed for more than 30 days after admission. The patients underwent laboratory and CT examination, and symptoms, treatments and outcome events were recorded after admission. The initial clinical data analyzed were as follows: age, sex, symptoms, underlying diseases, laboratory results and days from illness onset to admission. The endpoint of this study was the development of severe illness.

### CT examinations

All patients underwent chest CT examinations in the supine position by using the GE BrightSpeed Elite 16 scanner or GE LightSpeed VCT scanner (GE Healthcare, Milwaukee, USA) or Siemens SOMATOM Definition AS+ scanner (Siemens Healthineers, Milwaukee, Germany). The scanning range was from the apex to the bottom of the lung. The scanning parameters were as follows: tube voltage 120 kV, tube current automatic mA, helical pitch 0.938, rotation speed 0.6 s, slice thickness and spacing 5 mm. CT images were reconstructed with a slice thickness of 0.625–1.25 mm using a lung kernel as part of the reconstruction process.

### CT image analysis

All images were reviewed by two chest radiologists with 5–15 years of experience by consensus. Further review was undertaken by a third radiologist with 20 years of experience if there was disagreement. The initial CT images were evaluated for each of the 152 patients. The radiologists recorded the following lesion features: location, distribution, morphology, density, vascular bundle thickening, air bronchogram sign, crazy-paving sign, fibrosis, mosaic perfusion sign, pleural effusion, thoracic lymphadenopathy (defined as lymph node size of ≥10 mm in short-axis dimension), number of segments involved and “severity score of lung”. Location was recorded as unilateral lung or bilateral lung. Distribution was defined as peripheral, peripheral with central or central. Morphology was described as nodular or patchy, nodular with patchy or patchy with segmental. Density was recorded as ground glass opacity (GGO), GGO with consolidation or consolidation. Each of the five lung lobes was assessed for degree of involvement and scored as 0 (0% involvement), 1 (1–25% involvement), 2 (26–50% involvement), 3 (51–75% involvement), or 4 (76–100% involvement) [6]. An overall “severity score of lung” was calculated by summing the five lobe scores (range of scores, 0–20) [6].

### Statistical analysis

Categorical variables were reported as frequency and proportions, and continuous variables were reported as the mean ± standard deviation or median with interquartile range (IQR). The independent-sample t test or Mann-Whitney U test was performed to compare the quantitative parameters and Chi-square test to compare the qualitative features between the mild group and severe group. After potential risk factors were selected, multivariate logistic regression analysis was used to determine the independent risk factors associated with severe COVID-19. Selected variables were incorporated in the nomogram to predict severe COVID-19 using “rms” package in R software (version 3.5.1, http://www.rproject.org).

The performance of nomogram was assessed by discrimination and calibration. The discriminating ability of nomogram was evaluated using area under the receiver operating characteristic (ROC) curve (AUC). Calibration was evaluated using a calibration plot, a graphic representation of the relationship between the observed and predicted probability, with a bootstrapped sample of the study group for 1000 times. Further calibration of the nomogram was evaluated using the Hosmer-Lemeshow goodness of fit test. Decision curve analysis (DCA) was conducted to determine the clinical usefulness of the nomogram by quantifying the net benefits at different threshold probabilities and clinical impact curve to determine the influence on the outcome of patients. A risk chart plotted performance against caseload. The “rmda” package was used in DCA and “rattle” package in risk chart. Statistical analyses were performed with SPSS 20.0 and R software. All analyses were considered significant at *P* values of less than 0.05 (two-tailed).

## Results

### Clinical characteristics of patients with COVID-19

Of the 217 patients with COVID-19 included in this study, 212 patients have been discharged and 5 patients died. The majority of patients presented with fever, cough, sore throat or fatigue. A small number of patients had diarrhea or dyspnea. There were no significant differences in age, sex, clinical severity and basic disease between the training and validation cohorts (Table 1). Clinical characteristics of patients with COVID-19 in the training cohort was shown in Table 2. The mean age of the mild group was 40.70 years and the severe group was 56.18 years in the training cohort. The differences were statistically significant between the two groups (*P* < 0.05) (Table 2). Among the mild illness group, 13 (12.7%) patients had hypertension, 7 (6.9%) cases diabetes, 1(1.0%) coronary heart disease and 2 (2.0%) chronic obstructive pulmonary disease (COPD). For the severe illness group, 7 (14.0%) patients had hypertension, 14 (28.0%) diabetes, 4 (8.0%) coronary heart disease and 2 (4.0%) COPD. There was significant difference in diabetes and coronary heart disease (*P* < 0.05), but no significant difference in sex, hypertension and COPD between the two groups (*P* > 0.05) (Table 2). Blood leucocyte, neutrophil counts, platelet, neutrophil-to-lymphocyte ratio (NLR), platelet-to-lymphocyte ratio (PLR) and C-reactive protein (CRP) in the severe group were significantly higher than those in the mild group, and lymphocyte counts were significantly lower (*P* < 0.05) (Table 2). The median days from illness onset to admission were 4 days in the mild group and 5 days in the severe group, and the differences were not statistically significant (*P* > 0.05).

**Table 1:** Baseline characteristics of the study cohort

Clinical characteristics	Training cohort(*n* = 152)	Validation cohort(*n* = 65)	*t /* χ^2^ value	*P* value
Clinical severity			0.007	0.933
Mild illness	102 (67.1)	44 (67.7)		
Severe illness	50 (32.9)	21 (32.3)		
Age	45.79 ± 17.88	46.43 ± 16.45	0.248	0.805
Sex				
Male	87 (57.2)	40 (61.5)	0.347	0.556
Female	65 (42.8)	25 (38.5)		
Basic disease			2.778	0.096
Yes	23 (15.1)	16 (24.6)		
No	129 (84.9)	49 (75.4)		

Note.“Yes” of Basic disease means patients with one of the following disease: hypertension, diabetes, coronary heart disease, chronic obstructive pulmonary disease

Data are numbers of patients, with percentages in parentheses

**Table 2:** Clinical characteristics of patients with COVID-19 in the training cohort

Clinical characteristics	Mild group(*n* = 102)	Severe group(*n* = 50)	*t* / Z */* χ^2^ value	*P* value
Sex			0.691	0.406
Male	56 (54.9)	31 (62.0)		
Female	46 (45.1)	19 (38.0)		
Age	40.70 ± 17.21	56.18 ± 14.52	−5.476	< 0.001
Hypertension			0.046	0.830
Yes	13 (12.7)	7 (14.0)		
No	89 (87.3)	43 (86.0)		
Diabetes			12.59	0.001
Yes	7 (6.9)	14 (28.0)		
No	95 (93.1)	36 (72.0)		
Coronary heart disease			5.197	0.023
Yes	1 (1.0)	4 (8.0)		
No	101 (99.0)	46 (92.0)		
COPD			0.545	0.461
Yes	2 (2.0)	2 (4.0)		
No	100 (98.0)	48 (96.0)		
Leucocyte counts (×10^9^ /L)	5.11 (3.94–6.15)	6.31 (4.44–8.36)	−3.071	0.002
Neutrophil counts (× 10^9^ /L)	3.00 (1.94–3.89)	4.23 (3.00–6.57)	−4.471	< 0.001
Lymphocyte counts (×10^9^ /L)	1.33 (1.00–1.82)	0.91 (0.65–1.29)	−3.798	< 0.001
Platelet (×10^9^ /L)	178 (147–211)	204 (150–270)	−2.469	0.014
NLR	2.30 (1.41–3.22)	4.63 (2.67–7.34)	−5.749	< 0.001
PLR	132.64 (97.71–169.21)	219.37 (157.54–319.70)	−5.757	< 0.001
CRP (mg/L)	6.70 (0.90–17.60)	34.55 (7.48–70.52)	−5.419	< 0.001
Days from illness onset to admission	4 (2.0–6.0)	5 (3.0–7.0)	−1.259	0.208

*Note*: *COPD* chronic obstructive pulmonary disease, *NLR* neutrophil-to-lymphocyte ratio, *PLR* platelet-to-lymphocyte ratio, *CRP* C-reactive protein

Data are numbers of patients, with percentages in parentheses

### Initial CT characteristics in the training cohort

**Fig. 2:**
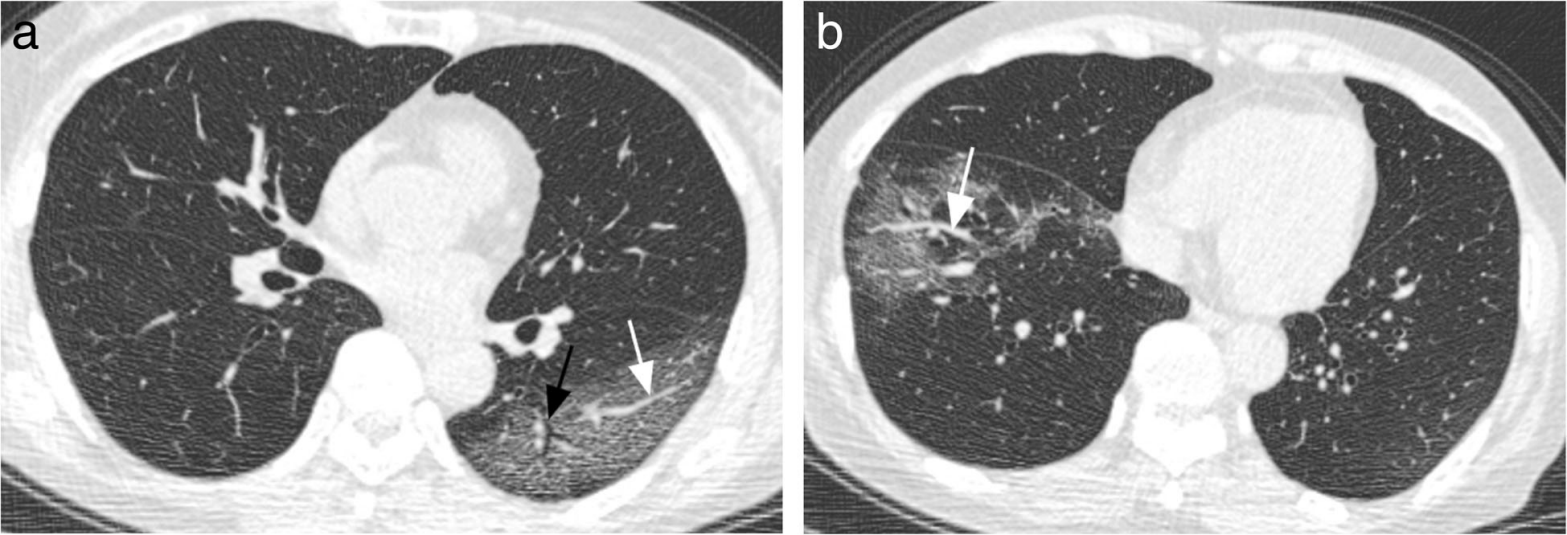
Chest CT images of a patient with mild COVID-19. **a** Axial CT images showed ground-glass opacity (GGO) in the left lower lobe. Vascular bundle thickening (white arrow), air bronchogram sign (black arrow) and crazy-paving sign were observed in the GGO. **b** Vascular bundle thickening (white arrow) was noted in the GGO of the right lower lobe

**Fig. 3:**
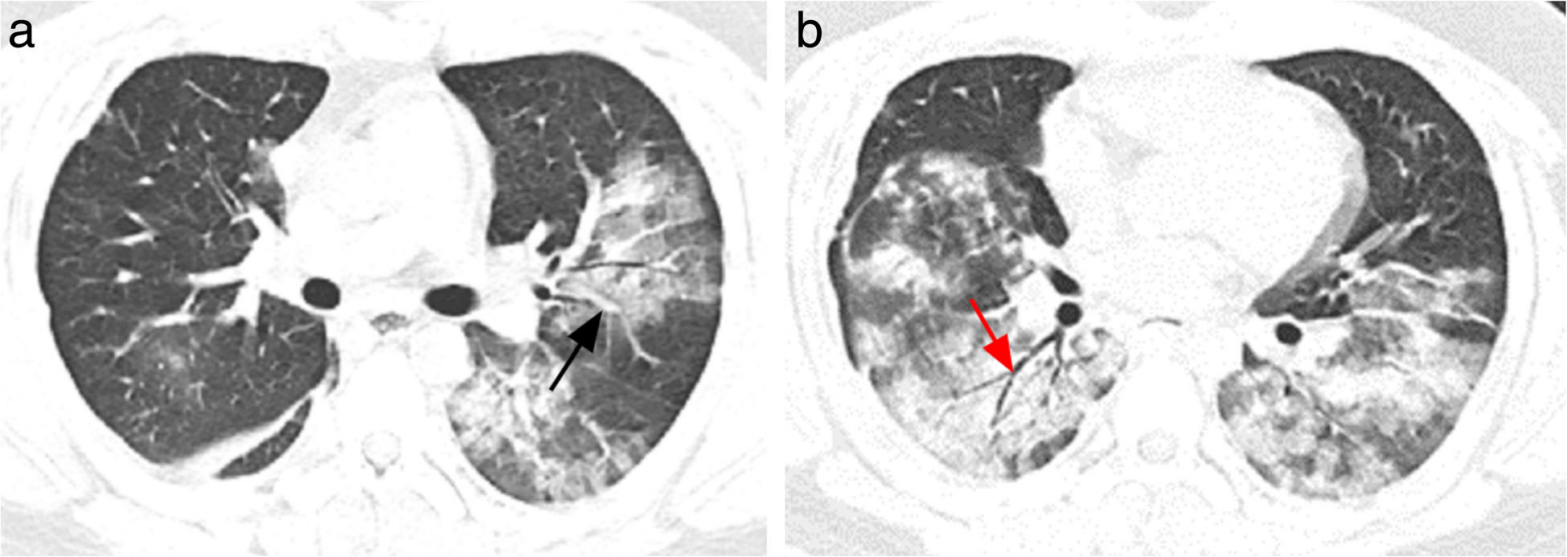
Chest CT images of a patient with severe COVID-19. **a-b** Axial CT images showed bilateral multifocal ground-glass opacity (GGO) with consolidation. Vascular bundle thickening (black arrow) and air bronchogram sign (red arrow) were observed in the COVID-19

**Fig. 4:**
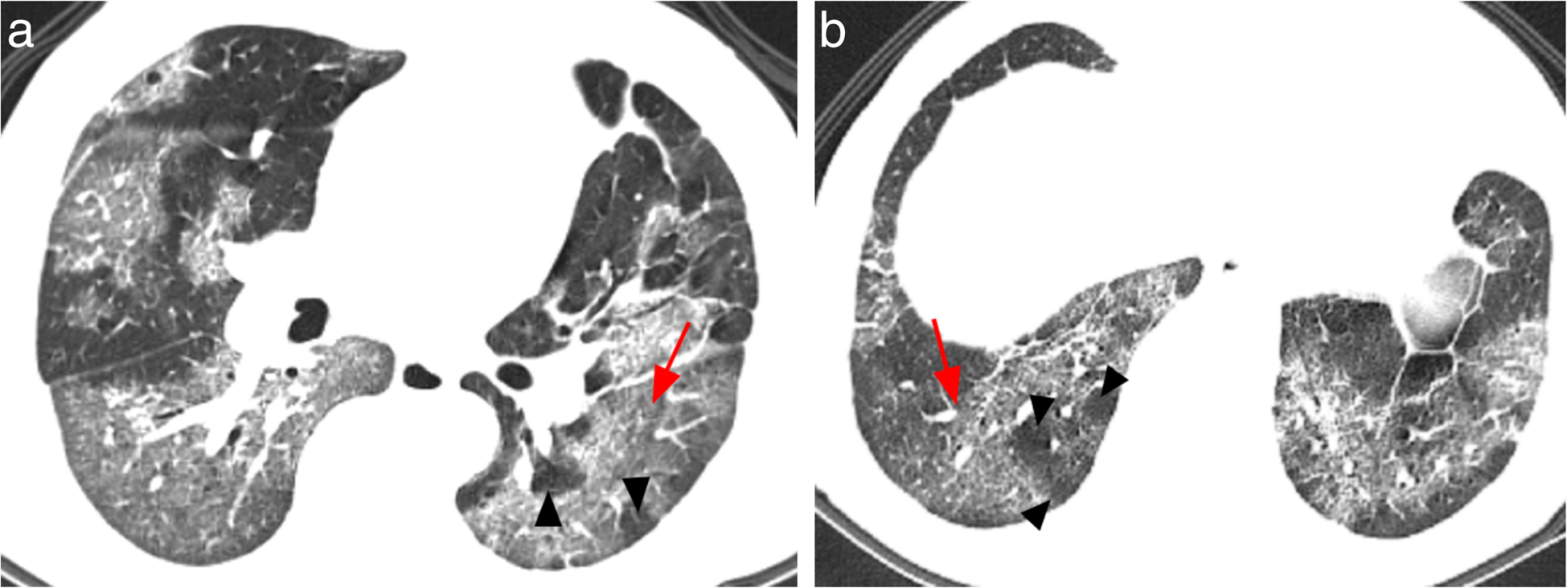
Chest CT images of a patient with severe COVID-19. **a-b** Axial CT images showed bilateral multifocal ground-glass opacity (GGO) in multiple lung segments. Mosaic perfusion sign composed of GGO (red arrow) and transparent shadow (black arrowhead) was present in the bilateral lower lobe

In the mild group, 38 patients (26.0%) had unilateral lung lesions and 108 patients (74.0%) showed bilateral lesions. In the severe group, 71 (100%) patients had bilateral lung lesions. In the training cohort, 74 patients (72.5%) presented bilateral lung lesions and 57 patients (55.9%) showed subpleural distribution in the mild group (Fig. 2). However, all patients (100.0%) were bilateral lung lesions and 43 patients (86.0%) had subpleural with central distribution in the severe group (Table 3) (Figs. 3 and 4). The density of lesions was mostly GGO or GGO with consolidation, and only consolidation was rare in the two groups. Vascular bundle thickening was seen in 69 patients (67.6%) of the mild group and 46 patients (92.0%) of the severe group. Crazy-paving sign was noted in 50 patients (49.0%) of the mild group and 37 patients (74.0%) of the severe group. Mosaic perfusion sign was observed in 1 patient (1.0%) of the mild group and 18 patients (36.0%) of the severe group (Table 3) (Fig. 4). There were significant differences in lesion location, distribution, morphology, density, vascular bundle thickening, air bronchogram sign, crazy-paving sign and mosaic perfusion sign (*P* < 0.05), but no significant differences in pulmonary fibrosis, pleural effusion and thoracic lymphadenopathy between the two groups (*P* > 0.05) (Table 3). Number of segments involved (10, IQR:9.0–11.0) and severity score of lung (7, IQR:5–9) in the severe group were significantly higher than those of the mild group [(5.5, IQR:3.0–8.0) vs (4, IQR:2–5)] (*P* < 0.001) (Table 3).

**Table 3:** Comparison of initial CT features between mild and severe patients in the train cohort

CT features	Mild group(*n* = 102)	Severe group (*n* = 50)	χ^2^ /Z Value	*P* Value
Location			16.828	< 0.001
Unilateral lung	28 (27.5)	0 (0.0)		
Bilateral lung	74 (72.5)	50 (100.0)		
Distribution			25.238	< 0.001
Subpleural	57 (55.9)	7 (14.0)		
Subpleural with central	44 (43.1)	43 (86.0)		
Central	1 (1.0)	0 (0.0)		
Morphology			31.109	< 0.001
Nodular or patchy	50 (49.0)	5 (10.0)		
Nodular with patchy	26 (25.5)	10 (20.0)		
Patchy with segmental	26 (25.5)	35 (70.0)		
Density			15.039	0.001
GGO	50 (49.0)	9 (18.0)		
GGO with consolidation	46 (45.1)	39 (78.0)		
Consolidation	6 (5.9)	2 (4.0)		
Vascular bundle thickening			10.805	0.001
Yes	69 (67.6)	46 (92.0)		
No	33 (32.4)	4 (8.0)		
Air bronchogram sign			5.834	0.016
Yes	40 (39.2)	30 (60.0)		
No	62 (60.8)	20 (40.0)		
Crazy-paving sign			8.554	0.003
Yes	50 (49.0)	37 (74.0)		
No	52 (51.0)	13 (26.0)		
Pulmonary fibrosis			1.314	0.252
Yes	45 (44.1)	27 (54.0)		
No	57 (55.9)	23 (46.0)		
Mosaic perfusion sign			37.621	< 0.001
Yes	1 (1.0)	18 (36.0)		
No	101 (99.0)	32 (64.0)		
Pleural effusion	7 (6.9)	5 (10.0)	0.125	0.723
Thoracic lymphadenopathy	18 (17.6)	10 (20.0)	0.124	0.725
Number of segments involved	5.5 (3.0–8.0)	10 (9.0–11.0)	−6.510	< 0.001
Severity score of lung	4 (2.0–5.0)	7 (5.0–9.0)	−7.712	< 0.001

*Note*: *GGO* ground glass opacity

Data are numbers of patients, with percentages in parentheses

### Nomogram construction and validation

**Fig. 5:**
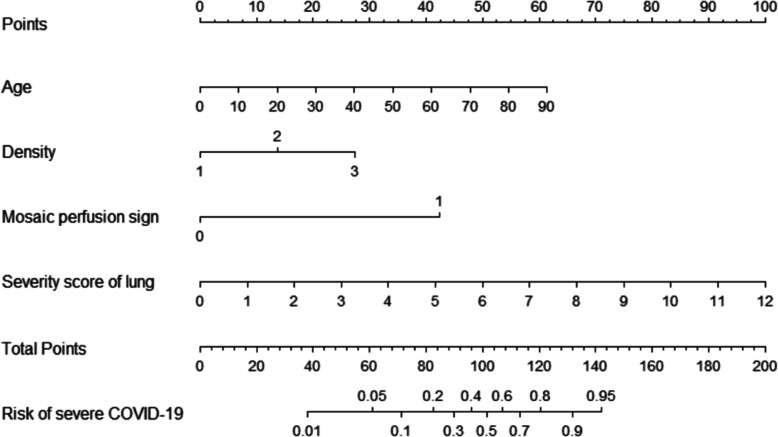
The nomogram was constructed to predict the risk of severe COVID-19 based on 4 independent risk factors. For binary variables, 0 = no and 1 = yes. For density category, 1 = GGO, 2 = GGO with consolidation, and 3 = consolidation. Abbreviations: GGO: ground glass opacity; COVID-19 = coronavirus disease 2019

**Fig. 6:**
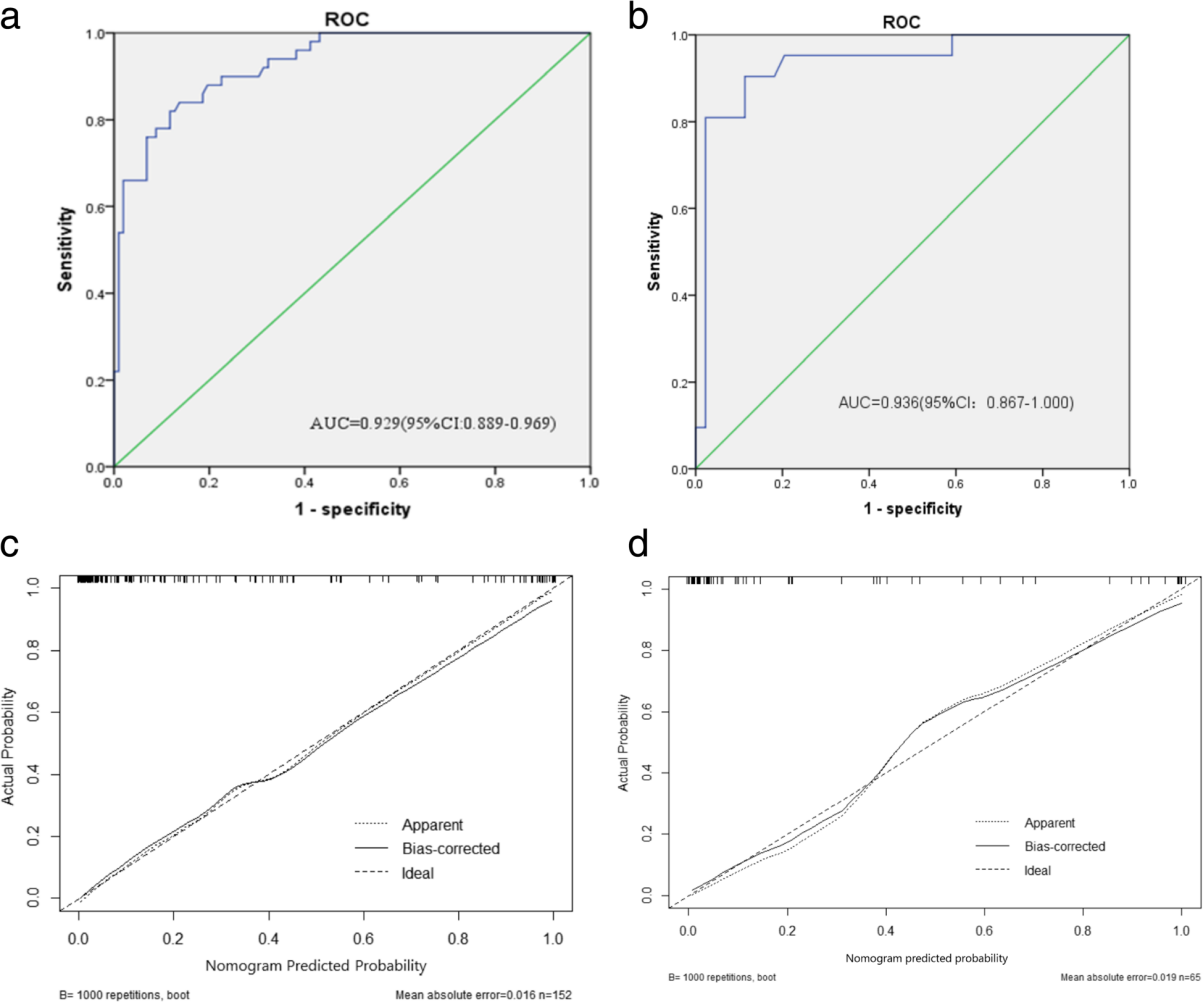
The ROC and calibration curves of the nomogram in the training cohort (**a, c**) and validation cohort (**b, d**), respectively. The y-axis of calibration curves represents the actual probability, the x-axis represents the predicted probability and the diagonal dashed line indicates the ideal prediction by a perfect model

**Fig. 7:**
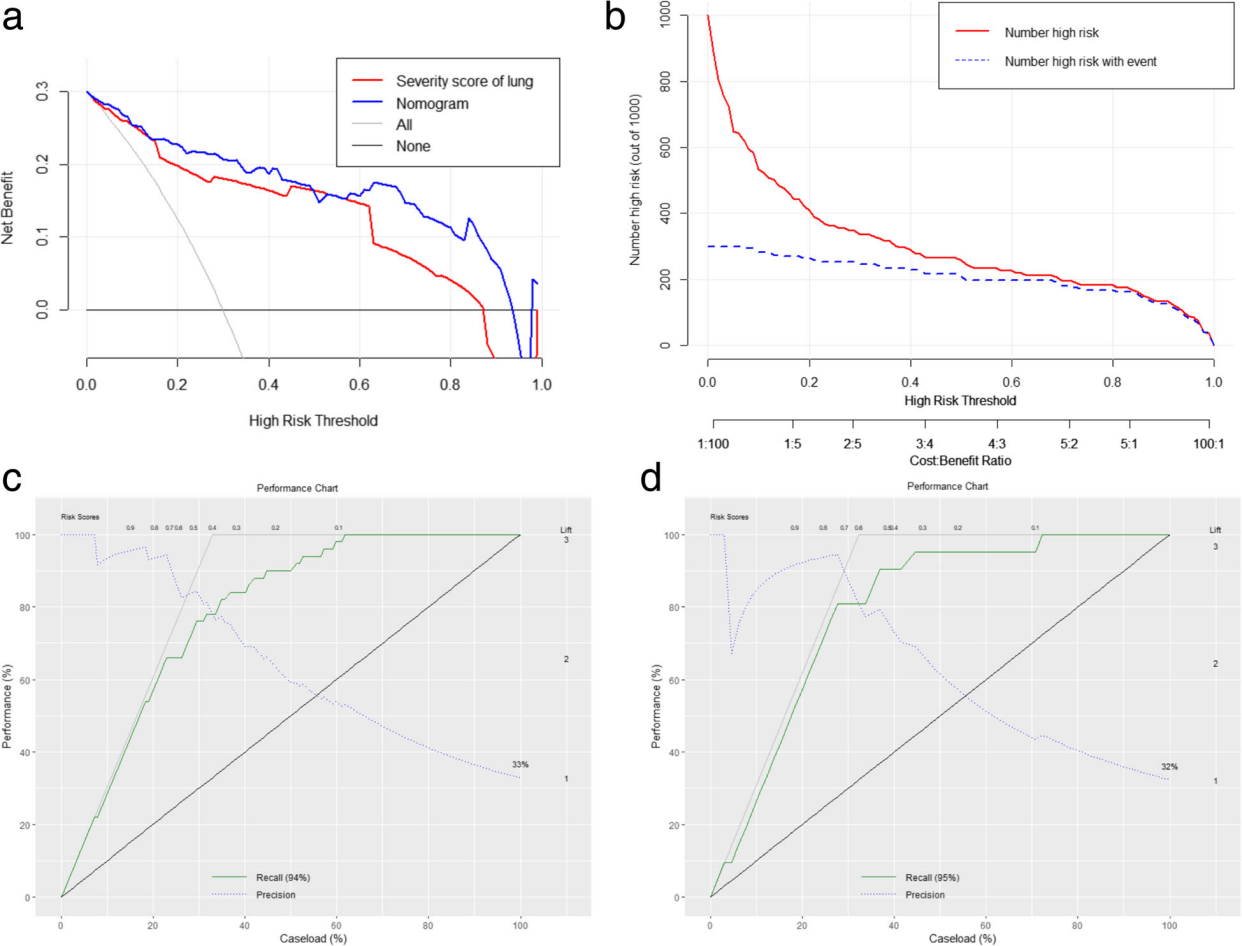
**a** Decision curve analysis for the nomogram. The decision curve indicated that when the threshold probability of a patient was within a range from 0.01 to 0.93, use of the nomogram for predicting severe COVID-19 would provide greater benefit than the “treat-all” or “treat-none” schemes. The curve of the nomogram over the severity score of lung showed the greatest benefit. **b** Clinical impact curve of the nomogram plotted the number of COVID-19 patients classified as high risk, and the number of cases classified high risk with severe COVID-19 at each high risk threshold. A risk chart plotted performance against caseload in the training cohort **c** and validation cohort **d**, respectively. Area under the recall (green) curves was 94 and 95% in the training and validation cohort, respectively

Multivariate logistic regression showed that age, density, mosaic perfusion sign and severity score of lung were independent risk factors for predicting severe patients based on the training cohort (Table 4). Then, a nomogram that incorporated the above independent predictors was developed (Fig. 5). The nomogram had a AUC of 0.929 (95% CI, 0.889–0.969), sensitivity of 84.0% (42/50) and specificity of 86.3% (88/102), in the training cohort, and a AUC of 0.936 (95% CI, 0.867–1.000), sensitivity of 90.5% (19/21) and specificity of 88.6% (39/44) in the validation cohort (Table 5; Fig. 6a and b). The calibration curves showed that the predicted probability was in highly agreement with the actual probability in the training and validation cohorts (Fig. 6c and d). The Hosmer-Lemeshow goodness of fit test yielded no significant difference between the predictive calibration curve and the ideal curve for predicting the severe patients both in the training (χ2 = 3.766, *P* = 0.878) and validation cohorts (χ2 = 7.843, *P* = 0.347). The decision curve showed that if the threshold probability was within a range from 0.01 to 0.93, more net benefit was added by using the nomogram for predicting severe patients than the “treat all” or “treat none” schemes (Fig. 7a). While the threshold probability was with a range from 0.03 to 0.87 for the severity score of lung. The clinical utility of the nomogram was the best (Fig. 7a). Clinical impact curve impacted the outcome of patients (Fig. 7b). A risk chart showed that area under the recall curves was 94 and 95% in the training and validation cohort, respectively (Fig. 7c and d).

**Table 4:** Multivariate logistic regression for predicting severe COVID-19

Variable	β	Odds ratio (95% CI)	*P*
Age	0.057	1.059 (1.008–1.112)	0.023
Diabetes	−0.771	0.463 (0.043–4.740)	0.523
Coronary heart disease	1.607	4.989 (0.221–112.387)	0.312
Leucocyte counts	−0.773	0.462 (0.076–2.803)	0.401
Neutrophil counts	0.956	2.601 (0.297–22.798)	0.388
Lymphocyte counts	0.036	1.036 (0.031–34.662)	0.984
Platelet	0.018	1.018 (0.984–1.053)	0.314
NLR	0.245	1.278 (0.432–3.780)	0.657
PLR	−0.007	0.993 (0.963–1.023)	0.633
CRP	−0.009	0.991 (0.974–1.008)	0.299
Location	9.328	2.508 (0.285–21.058)	0.395
Distribution	0.762	2.143 (0.32–14.366)	0.432
Morphology	−0.343	0.710 (0.238–2.121)	0.539
Density	1.546	4.694 (1.125–19.588)	0.034
Vascular bundle thickening	1.362	3.903 (0.515–29.559)	0.187
Air bronchogram sign	1.553	4.726 (0.785–28.457)	0.090
Crazy-paving sign	−0.414	0.661 (0.147–2.974)	0.590
Mosaic perfusion sign	5.562	260.314 (0.811–83,530.33)	0.039
Number of segments involved	−0.300	0.741 (0.531–1.035)	0.079
Severity score of lung	0.658	1.931 (1.100–3.391)	0.022

*Note*: *NLR* neutrophil-to-lymphocyte ratio, *PLR* platelet-to-lymphocyte ratio, *CRP* C-reactive protein, *β* regression coefficient

*P* < 0.05 indicates statistical significance

**Table 5:** Performance of nomogram for predicting severe COVID-19

Performance	Training cohort	Validation cohort
AUC (95% CI)	0.929 (0.889–0.969)	0.936 (0.867–1.000)
Accuracy	85.5% (130/152)	89.2%(25/65)
Sensitivity	84.0% (42/50)	90.5%(19/21)
Specificity	86.3% (88/102)	88.6%(39/44)

*Note*: *AUC* area under the receiver operating characteristic curve, *CI* confidence interval

## Discussion

COVID-19 is a new disease outbreak as a global health emergency, which has potentially far-reaching impact on public health. As is known to all, it is of great significance to predict the severity in the early stage of disease course. Liu et al. [9] reported that neutrophil-to-lymphocyte ratio (NLR) was a useful prognostic factor for severe COVID-19 incidence in the early stage. In the present study, a nomogram based on the clinical and CT imaging features of COVID-19 patients was developed and validated to predict the severity in the early stage. Our results demonstrated that age, density, mosaic perfusion sign and severity score of lung were independent risk factors for predicting severe patients and the nomogram might be a valuable tool for individual prediction of the incidence of severe COVID-19.

In our study, the patients with COVID-19 predominantly presented with fever, cough, sore or fatigue. It was consistent with the previous research results [1, 3, 11]. Older patients usually have more underlying diseases and lower immunity, and are more likely to become severe patients due to severe alveolar damage [12]. Patients with COVID-19 might show normal or lower leucocyte or lymphocyte counts, with increased CRP level [3, 9]. As shown in the present study, blood leucocyte and neutrophil counts in the severe group were significantly higher than those in the mild group. The increased leucocyte or neutrophil counts suggested the possible combination of bacterial infection due to low immune function [9]. Similar findings were made in the study by Wang D et al. [11]. NLR was a widely used marker for the assessment of the severity of bacterial infections [9, 13]. Platelet-to-lymphocyte ratio (PLR) has been described as a novel inflammatory marker, which may be used in many diseases for predicting inflammation and mortality [13]. Lee et al. [13] reported that NLR and PLR might be useful parameters in determining the severity of pneumonia. Liu et al. [9] indicated that the NLR was the most useful prognostic factor for severe illness patients with COVID-19. However, in our study, although NLR and PLR of the severe group were significantly higher than those of the mild group, NLR and PLR were only related factors, but not independent risk factors for predicting severe COVID-19. There might be two reasons for this. Firstly, NLR and PLR were calculated from the results of the blood tests in the patients who were initially admitted. Secondly, the CT features of patients were included in our study, but not in the study by Lee et al. [13] and Liu et al. [9].

In our study, CT imaging features of COVID-19 mainly included bilateral, multifocal and peripheral GGOs or GGOs with consolidations, which largely concurred with early studies [3, 6, 7]. Vascular bundle thickening, crazy-paving sign and air bronchogram sign were often seen in the mild and severe patients. The proportion of the severe patients with mosaic perfusion sign was significantly higher than that of the mild patients. This might be interpreted as that severe patients often had a large amount of fibromyxoid exudates in the alveolar or airway [12], resulting in gas retention due to sputum plug. Thus, mosaic perfusion sign appeared because of ventilation-perfusion abnormalities [12]. The severity score of lung in the severe group was significantly higher than that of the mild group in our study. It indicates that the range of pneumonia at initial chest CT is of great value in predicting the severe illness. As was reported by Xiong et al. [14] study about COVID-19, the severity of pneumonia assessed on initial CT were significantly related to the progression on follow-up CT.

Nomograms have frequently been used in the prognosis of the diseases, primarily for estimating the likelihood of an event [15, 16]. Liu et al. [9] reported the nomogram based on NLR had a c-index of 0.807 for predicting the severe COVID-19 probability. In the current study, we developed a nomogram model incorporating age, density, mosaic perfusion sign and severity score of lung. The nomogram model exhibited good predictive efficiency for severe COVID-19 in the training (AUC = 0.929) and validation cohorts (AUC = 0.936). The calibration curve, Hosmer-Lemeshow goodness of fit test and decision curve showed that the nomogram had high accuracy and superior net benefit. Thus, the nomogram can serve as a noninvasive predictive tool for assessment of the severity of patients with COVID-19.

Our study had several limitations. Firstly, the results were preliminary and need to be verified by additional studies performed with a larger number of samples. Secondly, the final survival outcome has not been included in the study. Future investigations are needed to draw broader conclusions. Thirdly, we focused on the early identification of severe patients based on initial clinical and CT characteristics. Hence, CT imaging features changes during follow-up were not included in our study.

## Conclusion

We used initial clinical and CT characteristics to develop and validate a nomogram for early prediction of the severity of patients with COVID-19. The nomogram offers clinicians a simple-to-use method for individualized evaluation of the patients with COVID-19, as well as making individualized decisions regarding the treatment.

## Supplementary information

**Additional file 1:.** Clinical severity and types of confirmed Coronavirus Disease 2019 (COVID-19).

## Data Availability

The datasets used and analyzed during the current study available from the corresponding author on reasonable request.

## Abbreviations

COVID-19: Coronavirus disease 2019
NLR: Neutrophil-to-lymphocyte ratio
PLR: Platelet-to-lymphocyte ratio
CRP: C-reactive protein
GGO: Ground-glass opacity
AUC: Area under the receiver operating characteristic curve
