# Supplementary material for: Nomogram to identify severe coronavirus disease 2019 (COVID-19) based on initial clinical and CT characteristics: a multi-center study
Source: BMC Med Imaging. 2020 Oct 2;20:111. doi: 10.1186/s12880-020-00513-z (PMC7530870; doi:10.1186/s12880-020-00513-z)
Supplement: Supplementary file 1 — Additional file 1:. Clinical severity and types of confirmed Coronavirus Disease 2019 (COVID-19). [file 12880_2020_513_MOESM1_ESM.docx]

Supplementary material

Clinical severity and types of confirmed Coronavirus Disease 2019 (COVID-19)

| Severity | Types | Findings |
| --- | --- | --- |
| Mild illness | Mild | Mild clinical symptoms without CT findings of pneumonia |
|  | Common | Fever and respiratory symptoms with CT findings of pneumonia |
| Severe illness | Severe | Meet any of the followings:  a. Respiratory distress, RR ≥30 times/min  b. SpO2 <93% at rest  c. PaO2/FiO2 ≤ 300 mmHg  d. Patients showing a rapid progression (>50%) on CT imaging within 24-48 hours |
|  | Critical | Meet any of the followings:  a. Respiratory failure, need mechanical ventilation  b. Shock  c. intensive care unit is required for combined organ failure |

Note: RR: respiratory rate; SpO2: oxygen saturation; PaO2: partial pressure of oxygen;

FiO2: fraction of inspired oxygen;

The clinical severity was divided into mild illness (mild and common types) and severe illness (severe and critical types) in our study.
